# Application of Nanotechnology in Cancer Diagnosis and Therapy - A Mini-Review

**DOI:** 10.7150/ijms.49801

**Published:** 2020-10-18

**Authors:** Cancan Jin, Kankai Wang, Anthony Oppong-Gyebi, Jiangnan Hu

**Affiliations:** 1 Department of Oncology, Affiliated Dongyang People's Hospital of Wenzhou Medical University, Dongyang, Zhejiang 322100,China; 2 Department of Neurosurgery, The First Affiliated Hospital of Wenzhou Medical University, Wenzhou, Zhejiang 325000, China; 3 Department of Pharmaceutical Sciences, University of North Texas Health Science Center, Fort Worth, Texas 76107, USA

**Keywords:** nanomaterials, nanotechnology, cancer, diagnosis, treatment

## Abstract

Cancer is a leading cause of death and poor quality of life globally. Even though several strategies are devised to reduce deaths, reduce chronic pain and improve the quality of life, there remains a shortfall in the adequacies of these cancer therapies. Among the cardinal steps towards ensuring optimal cancer treatment are early detection of cancer cells and drug application with high specificity to reduce toxicities. Due to increased systemic toxicities and refractoriness with conventional cancer diagnostic and therapeutic tools, other strategies including nanotechnology are being employed to improve diagnosis and mitigate disease severity. Over the years, immunotherapeutic agents based on nanotechnology have been used for several cancer types to reduce the invasiveness of cancerous cells while sparing healthy cells at the target site. Nanomaterials including carbon nanotubes, polymeric micelles and liposomes have been used in cancer drug design where they have shown considerable pharmacokinetic and pharmacodynamic benefits in cancer diagnosis and treatment. In this review, we outline the commonly used nanomaterials which are employed in cancer diagnosis and therapy. We have highlighted the suitability of these nanomaterials for cancer management based on their physicochemical and biological properties. We further reviewed the challenges that are associated with the various nanomaterials which limit their uses and hamper their translatability into the clinical setting in certain cancer types.

## Introduction

**Figure 1:**
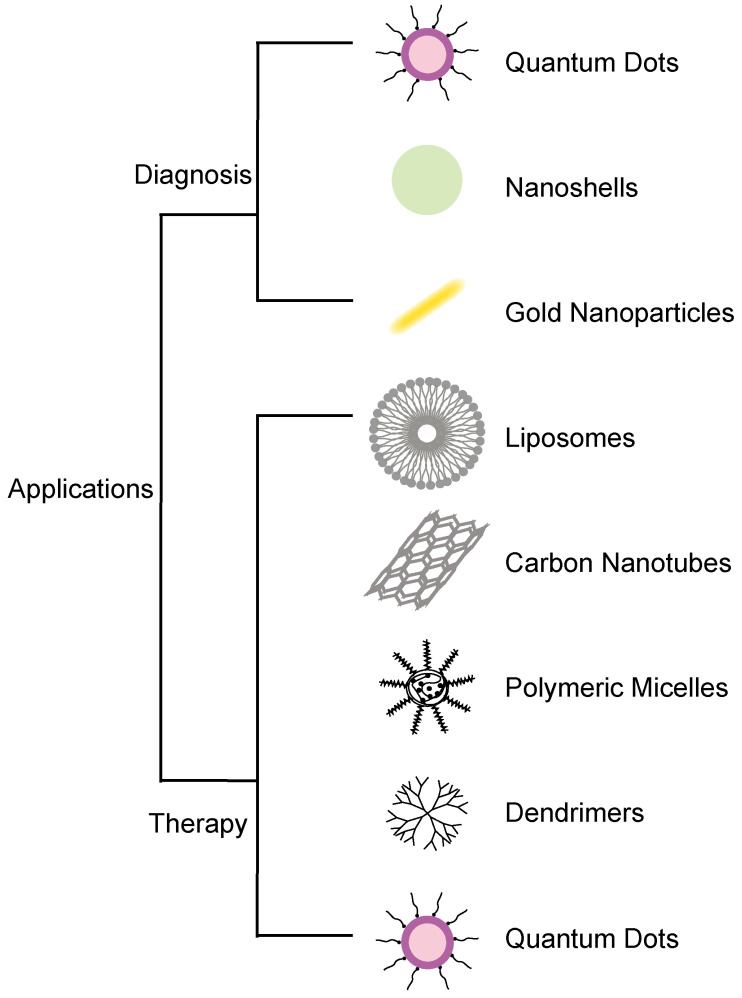
Application of nanomaterials in cancer diagnosis and therapy.

Cancer is a leading cause of death and a global health burden. It was estimated that there would be 18.1 million new cancer cases and 9.6 million cancer-related deaths by 2018^1^. Cancer is a disease characterized by uncontrolled cell proliferation that spreads from an initial focal point to other parts of the body to cause death. For these reasons, it is key to ensure earlier detection and treatment of cancers to reduce disease spread and mortalities. Amongst the widely used strategies, today in cancer research is nanotechnology. Nanotechnology has led to several promising results with its applications in the diagnosis and treatment of cancer, including drug delivery^2^, gene therapy, detection and diagnosis, drug carriage, biomarker mapping, targeted therapy, and molecular imaging. Nanotechnology has been applied in the development of nanomaterials^3^, such as gold nanoparticles and quantum dots, which are used for cancer diagnosis at the molecular level. Molecular diagnostics based on nanotechnology, such as the development of biomarkers, can accurately and quickly detect the cancers^4^. Nanotechnology treatments, such as the development of nanoscale drug delivery, can ensure precise cancerous tissue targeting with minimal side effects^5^, ^6^. Due to its biological nature, nanomaterials can easily cross cell barriers^7^. Over the years, nanomaterials have been used in the treatment of tumors, due to their active and passive targeting. Although many drugs can be used to treat cancers, the sensitivity of the drugs generally leads to inadequate results and can have various side effects, as well as damage to the healthy cells. In view of that, several studies have examined different forms of nanomaterials, such as liposomes, polymers, molecules, and antibodies, with the conclusion that a combination of these nanomaterials in cancer drug design can achieve a balance between increasing efficacy and reducing the toxicity of drugs^8^. However, due to the potential toxicity of nanomaterials, there is still a lot of advancement to be done on them before their readily acceptance in the clinic for cancer management^9^. With the rapid development of nanotechnology, this paper will review its application in cancer diagnosis and treatment with focus on their benefits and limitations during use **(Figure 1)**.

## Nanotechnology in Cancer Diagnosis

Genetic mutations can cause changes in the synthesis of certain biomolecules leading to uncontrolled cell proliferation and ultimately cancerous tissues^7^. Cancers can be classified as either benign or malignant. Benign tumors are confined to the origin of cancer while malignant tumors actively shed cells that invade surrounding tissues as well as distant organs. Cancer diagnostic and therapeutic strategies are targeted at early detection and inhibition of cancerous cell growth and their spread. Notable among the early diagnostic tools for cancers is the use of positron emission tomography (PET), magnetic resonance imaging (MRI), computed tomography (CT) and ultrasound^10^. These imaging systems, however, are limited by their inadequate provision of relevant clinical information about different cancer types and the stage. Hence it makes it difficult to obtain a full evaluation of the disease state based on which an optimum therapy can be provided 11, 12.

### Nanotechnology aids in tumor imaging

In the past few decades, the application of nanoparticles in cancer diagnosis and monitoring has attracted a lot of attention with several nanoparticle types being used today for molecular imaging. Due to their advantages including small size, good biocompatibility, and high atomic number, they have gained prominence in recent cancer research and diagnosis. Nanoparticles used in cancer such as semiconductors, quantum dots and iron oxide nanocrystals possess optical, magnetic or structural properties that are less common in other molecules 13. Different anti-tumor drugs and biomolecules including peptides, antibodies or other chemicals, can be used with nanoparticles to label highly specific tumors, which are useful for early detection and screening of cancer cells^14^.

For cancer diagnostics, imaging of tumor tissue with nanoparticles has made it possible to detect cancer in its early stages. In lung cancer, the detection of metastases can be determined by developing immune superparamagnetic iron oxide nanoparticles (SPIONs) that can be used in MRI imaging with the cancer cell lines as the target for the SPIONs 15. Recent studies have shown a high specificity of SPIONs with no known side effects, making them suitable building blocks for aerosols in lung cancer MRI imaging^16^-^18^,^19^.

Magnetic powder imaging has also been used in tomographic imaging technology where it has shown a high resolution and sensitivity to cancer tissues^20^. In animal experiments, nebulization of the lungs has been achieved using magnetic nanoparticles (MNPs) with Epidermal growth factor receptor (EGFR), a commonly expressed protein in non-small cell lung cancer (NSCLC) cases as a target. Further, *in vitro* studies using nanosystem for positron emission tomography (PET) have also been developed based on self-assembled amphiphilic dendritic molecules. These dendritic molecules spontaneously assemble into uniform supramolecular nanoparticles with abundant PET reporting units on the surface. By taking advantage of dendritic multivalence and the enhanced penetration and retention (EPR) effect, the dendritic nanometer system effectively accumulates in tumors, resulting in extremely sensitive and specific imaging of various tumors while reducing treatment toxicities.

### Nanotechnology Tools Used in Cancer Diagnosis

In current research, nanotechnology can validate cancer imaging at the tissue, cell, and molecular levels^20^. This is achieved through the capacity of nanotechnology applications to explore the tumor's environment, For instance, pH- response to fluorescent nanoprobes can help detect fibroblast activated protein-a on the cell membrane of tumor-associated fibroblasts^21^. Hereon, we will discuss some nanotechnology-based spatial and temporal techniques that can help accurately track living cells and monitor dynamic cellular events in tumors.

#### Near Infrared (NIR) Quantum Dots

The lack of ability to penetrate objects limits the use of visible spectral imaging. Quantum dots that emit fluorescence in the near-infrared spectrum (i.e., 700-1000 nanometers) have been designed to overcome this problem, making them more suitable for imaging colorectal cancer, liver cancer, pancreatic cancer, and lymphoma^22^-^24^. A second near-infrared (NIR) window (NIR-ii, 900-1700 nm) with higher tissue penetration depth, higher spatial and temporal resolution has also been developed to aid cancer imaging. Also, the development of a silver-rich Ag2Te quantum dots (QDs) containing a sulfur source has been reported to allow visualization of better spatial resolution images over a wide infrared range^25^.

#### Nanoshells

Another commonly used nanotechnology application is the use of nanoshells. Nanoshells are dielectric cores between 10 and 300 nanometers in size, usually made of silicon and coated with a thin metal shell (usually gold)^26^, ^27^. These nanoshells work by converting plasma-mediated electrical energy into light energy and can be flexibly tuned optically through UV-infrared emission/absorption arrays. Nanoshells are desirable because their imaging is devoid of the heavy metal toxicity^28^ even though their uses are limited by their large sizes.

#### Colloidal Gold Nanoparticles

**Figure 2:**
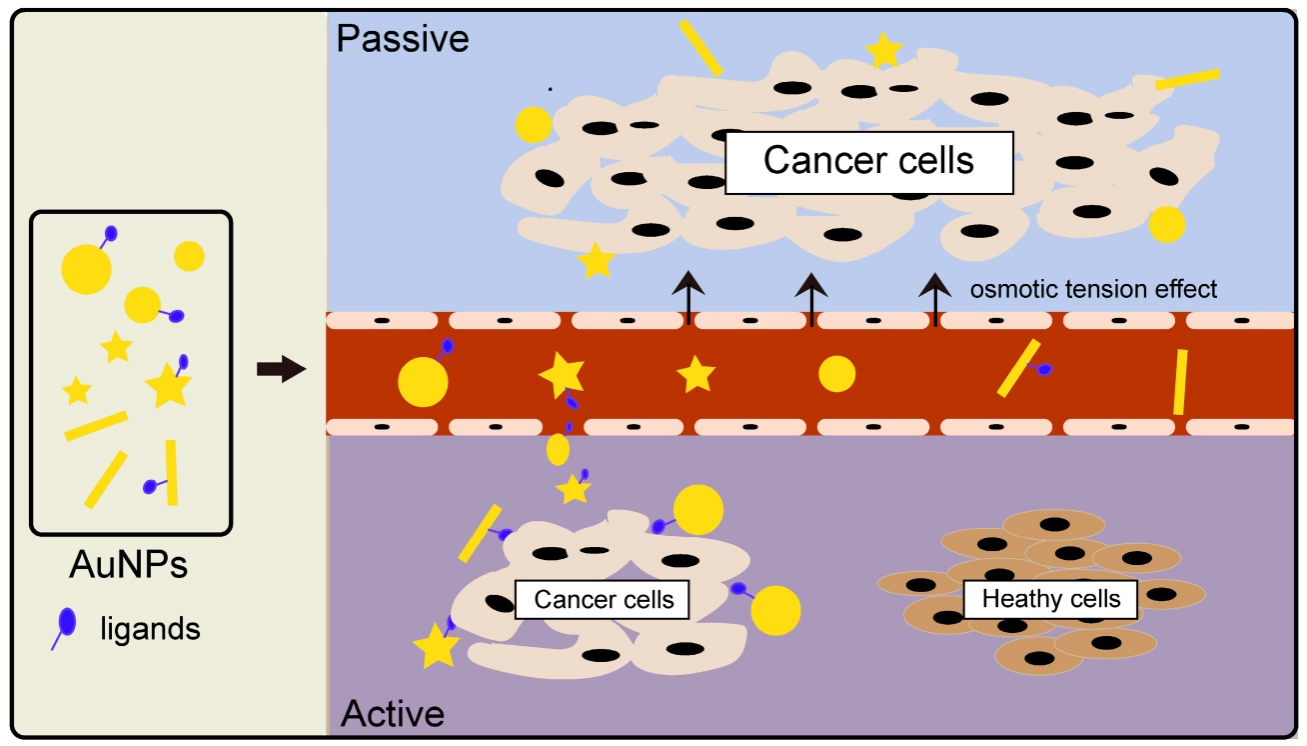
Various types of gold nanoparticles (different sizes, morphologies, and ligands) accumulate in tumor tissues by the action of osmotic tension effect (termed Passive targeting) or localize to specific cancer cells in a ligand-receptor binding way (termed Active targeting).

Gold nanoparticle (AuNPs) is a good contrast agent because of its small size, good biocompatibility, and high atomic number. Research shows that AuNPs work by both active and passive ways to target cells. The principle of passive targeting is governed by a gathering of the gold nanoparticles to enhance imaging because of the permeability tension effect (EPR) in tumor tissues^29^. Active targeting, on the other hand, is mediated by the coupling of AuNPs with tumor-specific targeted drugs, such as EGFR monoclonal antibodies, to achieve AuNP active targeting of tumor cells **(Figure 2)**. When the energy exceeds 80kev, the mass attenuation rate of gold becomes higher than alternative elements like iodine, indicating a greater prospect gold nanoparticles 30. Rand et al. mixed AuNPs with liver cancer cells and found that using X-ray imaging, the clusters of liver cancer cells in the gold nanocomposite group were significantly stronger than those in the liver cancer cells alone. These findings have important implications for early diagnosis, with the technique allowing tumors as small as a few millimeters in diameter to be detected in the body^31^.

### Nanotechnology used in cancer biomarker screening

Cancer biomarkers are biological features whose expression indicates the presence or state of a tumor. Such markers are used to study cellular processes, to monitor or identify changes in cancer cells, and these results could ultimately lead to a better understanding of tumors. Biomarkers can be proteins, protein fragments or DNA. Among them, tumor biomarkers, which are indicators of a tumor, can be tested to verify the presence of specific tumors. Tumor biomarkers ideally should possess a high sensitivity (>75%) and specificity (99.6%)^32^. Under current medical conditions, biomarkers from blood, urine, or saliva samples are used to screen individuals for cancer risk. But these biomarkers have not proven adequate for cancer screening. Therefore, several researchers have resorted to the study of extract patterns of abnormally expressed proteins, peptide fragments, glycans and autoantibodies from serum, urine, ascites or tissue samples from cancer patients^33^-^35^. With the development of proteomics technology, protein biomarkers for many cancers have been discovered.

In general, protein profiling tests would remove the high molecular weight proteins such as albumin and immunoglobulins. However, the removal of these proteins also removes the low molecular weight protein biomarkers conjugated to them, resulting in the loss of the biomarkers of interest. These low molecular weight proteins represent a potential biomarker-rich population^36^-^38^. Two studies led by Geho and Luchini came up with the method of capturing and enriching low molecular weight proteins by nanoparticles to obtain biomarkers from biological liquids, thus improving the screening of biomarkers^39^, ^40^. Nanoparticles compete with the carrier proteins by their surface characteristics, such as electric charge, or functional biomolecules, which are currently possessed by mesoporous silica particles, hydrogel nanoparticles, and carbon nanotubes^39^-^46^.

Another method to improve screening with nanocarrier is to improve the sensitivity of mass spectrometry. The unique optical and thermal properties of carbon nanotubes enhance the energy-transfer efficiency of the analyte, contributing to the absorption and ionization of the analyte, and eliminate the interference of inherent matrix ions^46^-^48^. A third approach is to use nanotechnology to make lab-on-chip microfluidics devices that can be used for immuno-screening or to study the properties of tumor cells. For example, a system showing great promise is lab-on-a-chip for high performance multiplexed protein detection using quantum dots made of cadmium selenide (CdSe) core with a zinc sulfide (ZnS) shell linked to antibodies to carcinoembryonic antigen, cancer antigen 125 and Her-2/Neu^49^. Another example is that cells growing on the surface of different sized nanometres, which were discovered by these nanometres across can differentiate between tumor cells^50^. Suffice it to say that there are still false-positive and false-negative results from screening of biomarkers by nanotechnology, and we need to improve sensitivity without compromising specificity.

## Nanotechnology in Cancer Therapy

### Tools of Nanotechnology for Cancer Therapy

The development of nanotechnology is based on the usage of small molecular structures and particles as tools for delivering drugs. Nano-carriers such as liposomes, micelles, dendritic macromolecules, quantum dots, and carbon nanotubes have been widely used in cancer treatment.

#### Liposomes

Liposomes are one of the most studied nanomaterials, which are nanoscale spheres composed of natural or synthesized phospholipid bilayer membrane and water phase nuclei^51^. Because of the amphiphilicity of phospholipids, liposomes form spontaneously^51^, allowing hydrophilic drugs to preferentially stay in the monolayer liposome while hydrophobic ones form before the multilayer liposome^52^. Some drugs could be incorporated into liposomes by exchanging them from acidic buffer to the neutral buffer. Neutral drugs can be transported in liposomes also, but due to a poor avidity for acidic environments, they are not readily released from the inside of the liposomes^53^. Other mechanisms of drug delivery are the combination of saturated drugs with organic solvents to form liposomes^51^. Under the influence of the EPR effect^53^, the vesicle of size around 4000 kDa or 500 nm can be allowed into the tumor by the gaps in vessels^52^. In tumors they can fuse with cells, are internalized by endocytosis, and release drugs in the intracellular space^52^. In the case of the appropriate pH, redox potential, ultrasonic and under the electromagnetic field, the liposome can also release the drug through passive or active ligand-mediated activity^52^. The targeted therapy has an advantage in the vascular system, micrometastases, and blood cancers^54^. It has been shown that the half-life of liposome is affected by size. The liposome up to 100 nanometers easily penetrate the tumor and stay longer, while the half-life of the bigger liposome is shorter because they are easily recognized and cleared by the mononuclear phagocyte system^55^. Liposome-bound antibodies target tumor-specific antigens to ensure active targeting and then transport drugs to the tumor. With a lot of pharmacokinetic benefits, some liposomal drugs are approved for clinical therapy **(Table 1)**. For instance, liposomal forms of adriamycin have been used for the management of metastatic ovarian cancer where they have shown appreciable clinical benefit^56^, ^57^.

#### Carbon Nanotubes

Based on the structure and the diameter, Carbon nanotubes (CNTs) can be categorized into two kinds, the single-walled CNTs (SWNTs) and the multiwalled CNTs (MWNTs)^58^. The SWNTs are composed of monolithic cylindrical graphene, and the MWNTs are composed of concentric graphene^58^. Because of the physical and chemical properties of carbon nanotubes, that include surface area, mechanical strength, metal properties, electrical and thermal conductivity, it is a candidate well suited for large-scale biomedical applications^59^. Carbon nanotubes also possess a property that allows them to absorb light from the near-infrared (NIR) region, causing the nanotubes to heat up by the thermal effect, hence can target tumor cells^60^-^62^. The natural forms of carbon nanotubes promote noninvasive penetration of biofilms and are regarded as highly competent carriers for the transport of various drug molecules into living cells^63^. Due to the suitability of carbon nanotubes, drugs such as paclitaxel are assembled with them and administered both *in vitro* and *in vivo* for cancer treatment^64^.

#### Polymeric Micelles

Polymeric nanoparticles (PNPs) are the inventions that relate to a solid micelle with a particle size range of 10-1000 nm^65^. PNPs are collectively known as polymer nanoparticle, nanospheres, nanocapsules or polymer micelles and they were the first polymers reported for drug delivery systems. PNPs serve as drug carriers for hydrophobic drugs and are widely used for drug discovery^66^-^68^. The PNPs constructed from amphiphilic polymers with a hydrophilic and hydrophobic block can perform rapid self-assembly because of the hydrophobic interactions in an aqueous solution^69^. The PNPs can capture the hydrophobic drugs because of a covalent bond or the interaction via a hydrophobic core. Thus, to carry the hydrophilic charged molecules, such as proteins, peptides, and nucleic acids, these blocks are switched to allow interactions in the core and neutralize the charge^67^.

The advantages of the higher thermodynamic stability and the smaller volume make the PNPs a suitable drug carrier with good endothelial cell permeability while avoiding kidney rejection^70^-^73^. The hydrophobic macromolecules and drugs can be transferred to the center of the PNPs, hence, the injection of PNPs suspension after being separated in an aqueous solution could achieve therapeutic effect^73^. Importantly, by oral or parenteral administration, drugs can reach the target cells in different ways, potentially provide alternative ways to lower cytotoxicity in healthy tissues compared to the cancer cells. However, the major challenges in the use of PNPs for cancer nanomedicine still exist in how to effectively deliver the drugs to the target site with limited side effects or drug resistance. Recently, the PNPs have been used widely in the nanotechnology-based cancer drug design due to their excellent potential benefits for patient care. For example, adriamycin conjugated nanomaterial was used to treat several types of cancers where it achieved therapeutic effects to a decent degree. However, it also presented with many side-effects, such as toxicity and heart problems, thereby limiting its use. Such problems are overcome by Doxil (a liposomal form of doxorubicin), which is less associated with cardiotoxicity in patients, and hence may provide a safer nanomaterial synthetic approach for researchers in the future^74^-^77^.

#### Dendrimers

The dendrimers are nanocarriers that have a spherical polymer core with regularly spaced branches^78^. As the dendritic macromolecule diameter increases, the tendency to tilt towards a spherical structure increases^79^. There are usually two ways to synthesize dendrimers, a divergent method in which the dendrimers can grow outward from the central nucleus, and a convergence method, where the dendrimers grow inward from the edges and end up in the central nucleus^80^, ^81^. Various molecules including polyacrylamide, polyglycerol-succinic acid, polylysine, polyglycerin, poly2, 2bis (hydroxymethyl) propionic acid, and melamine are commonly used to form dendrimers^82^. These dendritic macromolecules exhibit different chemical structures and properties, such as alkalinity, hydrogen bond capacity and charge, which can be regulated by growing dendritic macromolecules or changing the groups on the surface of dendritic macromolecules. In general, the dendritic drug conjugates are formed by the covalent binding of antitumor drugs to dendritic peripheral groups^83^. Thus, several drug molecules can attach to each dendritic molecule and the release of these therapeutic molecules is controlled in part by the nature of the attachment. The physicochemical and biological properties of the polymer including the size, charge, multi-ligand groups, lipid bilayer interactions, cytotoxicity, internalization, plasma retention time, biological distribution, and filtration of dendritic macromolecules, have made dendrimers potential nanoscale carriers^81^. Several studies have further shown that cancer cells with a high expression of folate receptors could form foils from dendritic molecules bound to folate^84^-^86^. An added advantage of dendrimers is their ability to bind to DNA as seen with the DNA-polyamides clustering DNA-poly(amidoamine) (DNAPAMAM), making them highly effective at killing cancer cells that express the folate receptor^87^.

#### Quantum Dots

Quantum dots (QDs) are small particles or nanocrystals of semiconductor materials between 2 and 10 nanometers in size^88^. The ratio of the height of the surface to the volume of these particles gives the QDs the intermediate electron property which is between a mass semiconductor and a discrete atom^89^. Over the years, various QDs based techniques such as modification of QD conjugates and QD immunostaining have been developed. With the improvement of multiplexing capability, QDs conjugation greatly exceeds the monochromatic experiment in both time and cost-effectiveness^90^. Moreover, at low protein expression levels and in a low context, QD immunostaining is more accurate than traditional immunochemical methods. In cancer diagnosis, QD immunostaining is a potential tool for the detection of various tumor biomarkers, such as a cell protein or other components of a heterogeneous tumor sample^91^. Quantum dots can gather in specific parts of the body and transfer the drugs to those parts. The ability of the QDs to concentrate in a single internal organ makes them a potential solution against untargeted drug delivery, and possibly avoid the side effects of chemotherapy. The latest advancement in surface modification of QDs, which combine with biomolecules, including peptides and antibodies, *in vivo*, can be used to target tumors and make possible their potential applications in cancer imaging and treatment. Some studies combine QDs with prostate-specific antigen to label cancer, while others use QDs to make biomarkers that speed up the process with such immune markers having a more stable light intensity than traditional fluorescent immunomarkers^92^. High sensitivity probes based on quantum dots have been reported for multicolor fluorescence imaging of cancer cells *in vivo* and can also be used to detect ovarian cancer marker cancer antigen 125 (CA125) in different types of specimens (such as fixed cells, tissue sections, and xenograft) 93. Besides, the light stability of quantum dot signals is more concrete and brighter than that of traditional organic dyes^94^. Chen et al. successfully detected BC using quantum-dot-based probes, confirming that unlike traditional immunohistochemistry, quantum dot immunohistochemistry (IHC) can detect the very low expressions of Human Epidermal Growth Factor Receptor 2 (HER2) as well as multichannel detection^95^, ^96^.

## Conclusion and Future directions

Nanotechnology has shown a lot of promise in cancer therapy over the years. By their improved pharmacokinetic and pharmacodynamic properties, nanomaterials have contributed to improved cancer diagnosis and treatment. Nanotechnology allows targeted drug delivery in affected organs with minimal systemic toxicities due to their specificities. However, as with other therapeutic options, nanotechnology is not completely devoid of toxicities and comes with few challenges with its use including systemic and certain organ toxicities, hence, causing setbacks with their clinical applications. Given the limitations with nanotechnology, more advancements must be done to improve drug delivery, maximize their efficacy while keeping the disadvantages to the minimum. By improving the interactions between the physicochemical properties of the nanomaterials employed, safer and more efficacious derivatives for diagnosis and treatment can be made available for cancer management. In sum, we sought to highlight the key advantages of nanotechnology and the shortfalls in their use to meet clinical needs for cancer. Adding to that, the therapeutic benefits of nanotechnology and future advancements could make them a therapeutic potential to be applied in other disease conditions. These may include ischemic stroke and rheumatoid arthritis which would require targeted delivery of a suitable pharmacologic agent at the affected site.

## Figures and Tables

**Table 1:** Nanomaterial-carrying drugs in clinical trials of cancer treatment in the past five years.

	Year	Drugs	Disease	Findings	Reference
Liposome	2015	Doxorubicin	Platinum-Sensitive Ovarian Cancer	favorable risk-benefit profile	97
		Paclitaxel	Non-Small Cell Lung Cancer	considerable disease response and resection rate, with acceptable toxicity	98
		Ursolic acid	Advanced Solid Tumors	tolerable, manageable toxicity, improving patient remission rates	99
		Mitomycin C	advanced cancer	long circulation time, tolerable, effective	100
	2016	miR-34a Mimic	Advanced Solid Tumors	effective	101
		Vincristine Sulfate	Refractory Solid Tumors or Leukemias	without dose-limiting neurotoxicity	102
		5-fluorouracil and Leucovorin	Advanced Solid Tumors	lower peak plasma concentration, longer half-life, and increased area	103
		Cytarabine	Childhood Acute Lymphoblastic Leukemia	no permanent adverse neurological sequelae	104
	2017	Amphotericin	Acute Lymphoblastic Leukaemia	effective	105
		Irinotecan	Recurrent High-Grade Glioma	no unexpected toxicities	106
	2018	Cytarabine and Daunorubicin	Newly Diagnosed Secondary Acute Myeloid Leukemia	significantly longer survival rate	107
		Curcumin	Locally Advanced or Metastatic Cancer	durable	108
		Daunorubicin	Pediatric Relapsed/Refractory Acute Myeloid Leukemia	well-tolerated and showed high response rates	109
		Lipovaxin-MM	Malignant Melanoma	well-tolerated and without clinically significant toxicity	110
		Vincristine Sulfate	Acute Lymphoblastic Leukemia	provided a meaningful clinical benefit and safety	111
		Oligodeoxynucleotide	Refractory or Relapsed Haematological Malignancies	well-tolerated, effective	112
	2019	Eribulin	Solid Tumours	well-tolerated with a favorable pharmacokinetic profile	113
Polymeric Micelles	2017	Epirubicin	Solid tumors	Well tolerated in patients with various solid tumors and exhibited less toxicity than conventional epirubicin formulations	114
	2018	Genexol-PM plus carboplatin	Ovarian Cancer	Non-inferior efficacy and well-tolerated toxicities	115
	2019	Paclitaxel (PTX)	Breast cancer	NK105 had a better PSN toxicity profile than PTX	116

## Abbreviations

AuNPs: Gold Nanoparticles
BC: Breast Cancer
CA125: Cancer Antigen 125
CdSe: cadmium selenide
CNTs: Carbon Nanotubes
CT: Computed Tomography
DNAPAMAM: DNA-poly(amidoamine)
EGFR: Epidermal Growth Factor Receptor
HER2: Human Epidermal Growth Factor Receptor 2
IHC: Immunohistochemistry
MNPs: Magnetic Nanoparticles
MWNTs: Multiwalled Carbon Nanotubes
MRI: Magnetic Resonance Imaging
NIR: Near Infrared
NSCLC: Non-small Cell Lung Cancer
nm: nanometers
PET: Positron Emission Tomography
PNPs: Polymeric Nanoparticles
QDs: Quantum Dots
SPION: Superparamagnetic Iron Oxide Nanoparticles
SWNTs: Single-walled Carbon Nanotubes
ZnS: zinc sulfifide
